# Complete mitochondrial genome of *Tachysurus vachellii*, natural diploid catfish from Nansi Lake

**DOI:** 10.1080/23802359.2021.1915714

**Published:** 2021-09-22

**Authors:** Ming Zhang, Chunqiao Zhao, Lei Song, Chao Sun, Yuxuan Ma, Qiang Liu, Yiming Yu, Guosong Zhang, Guisheng Zhang, Haili Zhang

**Affiliations:** a Key Laboratory for Physiology Biochemistry and Application, Heze University, Heze, China; b Nantong Xuyang Biological Technology Co. Ltd., Nantong, China; c Traditional Chinese Medicine Hospital of Dingtao District, Heze, China

**Keywords:** *Tachysurus vachellii*, mitogenome, phylogenetic analysis

## Abstract

*Tachysurus vachellii* are commercially important edible fish due to delicious taste, little bone in muscle, and high nutritional value especially in Asia. The complete mitochondrial genome of *Tachysurus vachellii* has been sequenced. The mitochondrial genome is 16,529 bp in length, with the base composition of 31.6% A, 26.6% T, 26.9% C, and 14.9% G, containing two ribosomal RNA genes, 13 protein-coding genes, 22 transfer RNA genes, and a major non-coding control region (D-loop region). The gene order and orientation are similar with some typical fish species. The data will provide useful molecular information for phylogenetic studies concerning *T. vachellii* and its related species.

*Tachysurus vachellii* are commercially important edible fish due to delicious taste, little bone in muscle, and high nutritional value especially in Asia (Zhang et al. 2016a). However, due to overfishing, environmental pollution and other factors, the capture production of *T. vachellii* has been declining in the recent years (Zhang et al. 2016b). To benefit the sustainable utilization of *T. vachellii* fishery resource, it is necessary to determine the complete mitogenome sequence and make clear its phylogenetic relationships with closely related species. This information can also provide a theoretical basis for the studies on molecular systematics, stock evaluation, conservation genetics, and evolutionary adaptation mechanisms (Chak et al. 2020).

In the present study, the biological specimen was collected from Nansi Lake, north latitude 34°66″ and east longitude 117°20″, Jining city, China. They were preserved in 95% alcohol, which were stored in biology herbarium of Heze University under the voucher number: Zhangming214. All DNA were extracted using phenol–chloroform extraction methods and stored at −80 °C. The mitogenome was amplified by primers which were initially published (Zhang et al. 2015). Fragments generated from PCR amplification were sequenced using Sanger sequencing technology (Yuping et al. 2019). Sequenced fragments were assembled to create the complete mitogenome using CodonCode Aligner 5.1.5 (CodonCode Corporation, Dedham, MA), followed with sequence analysis, assembly, and visualization using SepMan and DNAMan (Wang et al. 2021). The complete mitogenome was annotated using the software of Sequin (version 15.10, http://www.ncbi.nlm.nih.gov/Sequin/). Transfer RNA genes and their potential cloverleaf structures were identified using tRNAscan-SE 1.21. All newly determined sequences from this study were deposited in GenBank database (accession number: MW288250).

The total length of *T. vachellii* mitochondrial DNA is 16,529 bp, which includes 13 protein-coding genes, 22 tRNA genes, two rRNA genes, and two non-coding regions: a putative control region (D-loop region) and the origin of light-strand replication. All genes showed the typical gene arrangement conforming to the vertebrate consensus (Prosdocimi et al. 2012). The content is 31.6% for A, 26.6% for T, 26.9% for C, and 14.9% for G. A high A + T content indicates an obvious antiguanine bias commonly observed in fishes (Qiao et al. 2013). Except for eight tRNA (*Gln, Ala, Asn, Cys, Tyr, Ser, Glu*, and *Pro*) genes and ND6, most of the genes were encoded on the heavy strand (H-strand). All genes displayed the typical gene arrangement conforming to the vertebrate consensus (Chen et al. 2013). In the 13 protein-coding genes of mitochondrial genome, four overlaps are detected as shown in: *ATP8-ATP6*, *ATP6-COXIII*, *ND4-ND4L*, and *ND5-ND6* sharing 10, 1, 7, and 4 nucleotides, respectively. The overlap of the ATPase genes appears to be common in most vertebrate mitochondrial genome (7–10 bp) (Broughton et al. 2001). Among the 22 tRNA genes, three overlaps are found, i.e. *tRNA^Ile^-tRNA^Gln^*, *tRNA^Gln^-tRNA^Met^*, and *tRNA^Thr^-tRNA^Pro^*. There are also two overlaps between *ND2* and *tRNA^Trp^* and between *ND3* and *tRNA^Arg^*. The D-loop was 887 bp, flanked by *tRNA^Pro^* and *tRNA^Phe^* genes.

The maximum-likelihood tree by MEGA 7 based on 13 protein coding genes of the *T. vachellii* and the other 12 kinds of fish were constructed (Park et al. 2019). Seen from the phylogenetic tree (Figure 1), *T. vachellii* have a closer relationship with *Tachysurus eupogon*. It is worth noting that a member of same genus, *T. fulvidraco*, was independent for one branch, which may be one of the reasons of mis-identification. Pseudobagrus, Leiocassis, Tachysurus clustered into Bagridae were the closest kinship groups. Compared with above groups, Hemibagrus was an early differentiation genus, followed by Pangasiidae. Above branches and Bagridae formed the sister group. Finally, they formed a sister group with Sisoridae.

**Figure 1.**
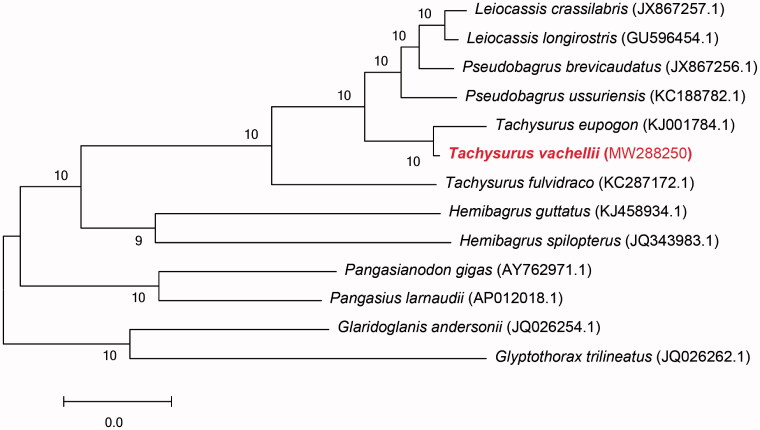
Maximum-likelihood tree based on mitochondrial genome nucleotide sequences of the *Tachysurus vachellii* and the other 12 kinds of fish. *Tachysurus vachellii* (MW288250) in the position of the evolutionary tree. Numbers above branches are bootstrap values by 1000 replicates. The GenBank accession numbers of the sequences for the other 12 kinds of fish were used in the tree.

## Data Availability

The data that support the findings of this study are openly available in GenBank of National Center for Biotechnology Information at https://www.ncbi.nlm.nih.gov, reference number MW288250.

## References

[CIT0001] Broughton RE, Milam JE, Roe BA. 2001. The complete sequence of the zebrafish (*Danio rerio*) mitochondrial genome and evolutionary patterns in vertebrate mitochondrial DNA. Genome Res. 11:1958–1967. 11691861 10.1101/gr.156801 PMC311132

[CIT0002] Chak STC, Barden P, Baeza JA. 2020. The complete mitochondrial genome of the eusocial sponge-dwelling snapping shrimp *Synalpheus microneptunus*. Sci Rep. 10:47285. 10.1038/s41598-020-64269-w PMC7210941 32385299

[CIT0003] Chen Y, Cheng Q, Qiao H, Zhu Y, Chen W. 2013. The complete mitochondrial genome sequence of *Rastrelliger kanagurta* (Perciformes: Scombridae). Mitochondrial DNA. 24:114–116. 23025587 10.3109/19401736.2012.726624

[CIT0004] Park J, Kim Y, Xi H. 2019. The complete mitochondrial genome sequence of Chinese minnow in Korea, *Rhynchocypris oxycephalus* (Sauvage and Dabry de Thiersant, 1874). Mitochondrial DNA B. 4:662–663.

[CIT0005] Prosdocimi F, Carvalho DCD, Beheregaray ALB. 2012. The complete mitochondrial genome of two recently derived species of the fish genus *Nannoperca* (Perciformes, Percichthyidae). Mol Biol Rep. 39:2767–2772. 21681429 10.1007/s11033-011-1034-5

[CIT0006] Qiao H, Cheng Q, Chen Y, Chen W, Zhu Y. 2013. The complete mitochondrial genome sequence of *Coilia ectenes* (Clupeiformes: Engraulidae). Mitochondrial DNA. 24:123–125. 23072559 10.3109/19401736.2012.731405

[CIT0007] Wang Y, Du Y, Song X. 2021. Complete mitochondrial genome sequence of *Anax parthenope* (Odonata: Anisoptera: Aeshnidae) and phylogenetic analysis. Mitochondrial DNA B. 6(1):122–123. 10.1080/23802359.2020.1848479 PMC7808743 33490598

[CIT0008] Yuping L, Xu S, Susup S, Lvsup T, Tao L. 2019. Characterization of the complete chloroplast genome sequence of *Littledalea racemosa* Keng (Poaceae: Bromeae). Mitochondrial DNA B. 13:33–35.

[CIT0009] Zhang G, Mao J, Liang F, Chen J, Zhao C, Yin S, Wang L, Tang Z, Chen S. 2016a. Modulated expression and enzymatic activities of Darkbarbel catfish, *Pelteobagrus vachelli* for oxidative stress induced by acute hypoxia and reoxygenation. Chemosphere. 151:271–279. 26945243 10.1016/j.chemosphere.2016.02.072

[CIT0010] Zhang G, Wang R, Mao J, Yin S, Chen S. 2015. The complete mitochondrial genome and phylogenic analysis of *Pseudobagrus vachelli*. Mitochondrial DNA. 27:1–2. 10.3109/19401736.2015.1074213 26260177

[CIT0011] Zhang G, Yin S, Mao J, Liang F, Zhao C, Li P, Zhou G, Chen S, Tang Z. 2016b. Integrated analysis of mRNA-seq and miRNA-seq in the liver of *Pelteobagrus vachelli* in response to hypoxia. Sci Rep. 6:22907. 26961594 10.1038/srep22907 PMC4785494

